# Modeling Economic Effects of Vaccination Against Porcine Reproductive and Respiratory Syndrome: Impact of Vaccination Effectiveness, Vaccine Price, and Vaccination Coverage

**DOI:** 10.3389/fvets.2020.00500

**Published:** 2020-08-11

**Authors:** Beat Thomann, Jonathan Rushton, Gertraud Schuepbach-Regula, Heiko Nathues

**Affiliations:** ^1^Department of Clinical Research and Veterinary Public Health, Vetsuisse Faculty, Veterinary Public Health Institute, University of Bern, Bern, Switzerland; ^2^Institute of Infection and Global Health, University of Liverpool, Liverpool, United Kingdom; ^3^Department of Clinical Veterinary Medicine, Vetsuisse Faculty, Clinic for Swine, University of Bern, Bern, Switzerland

**Keywords:** economic modeling, pig production, disease intervention, PRRS control, vaccination effectiveness

## Abstract

Porcine reproductive and respiratory syndrome (PRRS) causes substantial financial losses in pig farms and economic losses to societies worldwide. Vaccination against PRRS virus (PRRSV) is a common intervention in affected farms. The aim of this study was to assess the economic impact and profitability of potential new PRRS vaccines with improved efficacy at animal, herd, and national level. Two vaccination strategies were modeled; (i) mass vaccination of sows only (MS) and (ii) mass vaccination of sows and vaccination of piglets (MSP), comprising different scenarios of vaccine effectiveness, vaccine price, and vaccination coverage. A farrow-to-finish farm with 1,000 working sows from a pig-dense region in Germany served as an example farm. Financial benefits were obtained from gross margin analyses and were defined as difference in gross margin between a PRRSV-infected farm without vaccination (baseline) and with vaccination (intervention). Financial benefits were highest if both sows and piglets (MSP) were vaccinated. In these scenarios, median annual net benefits per working sow ranged from €170 to 340. If sows only were vaccinated (MS), estimated benefits attributable to vaccination were between €148 and 270. Decisive variables for the estimation of national level benefits were the number of farmers switching from existing to a better protecting vaccine, the number of previously non-vaccinating herds adopting the new vaccine, and the effectiveness of the new vaccine relative to those already available. Benefits were greatest when the new vaccine was adopted by previously non-vaccinating herds. The analyses showed that vaccination against PRRS was beneficial for all modeled scenarios. The magnitude of benefits derived from vaccination was more susceptible to changes in vaccination effectiveness than to vaccine price changes. This study provides evidence to support future vaccine development. The estimates indicate that the introduction of more efficient vaccines might lead to substantial financial benefits, is of socio-economic importance and that new vaccines might significantly contribute to the reduction of disease burden.

## Introduction

Porcine reproductive and respiratory syndrome (PRRS) causes substantial economic losses in pig production worldwide (1–4). In the United States, the estimated annual cost of PRRS on the national level was estimated at US $664 million, based on a population size of 5.8 million breeding sows and 110 million pigs marketed per year (2). At farm level, costs due to PRRS depend on the individual farm situation including factors such as disease severity, affected production stages, or farm size. Median losses in a farrow-to-finish farm in Germany with 1,000 working sows moderately affected in all production stages were estimated at € 442′973 per year (3). Both the reproduction component of the disease manifested in breeding sows and the respiratory component in growing pigs contribute substantially to the economic losses (2, 3).

Various strategies have been developed to control PRRS. Apart from elimination procedures with the aim to create a completely virus-negative herd (e.g., closure and roll-over) or improvement of biosecurity and management, vaccination against PRRS is a common approach to control the disease (5). Depending on individual farm situation, several intervention strategies have shown to be profitable, with mass vaccination of sows or mass vaccination of sow and piglets being amongst the most cost efficient control strategies (6).

Besides the commercially available inactivated vaccines and modified-live virus vaccines (MLV), new techniques for administration have been explored and developed and research on the use of DNA vaccines to further improve efficacy of MLV vaccines has been conducted (7–9). However, current vaccines fail to provide complete protection against heterologous field strains and commercially available vaccines only partially prevent or mitigate the disease and show limited effectiveness in the field (10–13).

Most literature on vaccine efficacy describes effects under ideal study conditions. Typically, vaccine efficacy is assessed in experimentally infected pigs and refers to clinical, virological, or pathological findings after challenge under experimental conditions. Outcomes are reported as reduction of viremia in terms of duration and/or magnitude, of clinical signs or of pathological lesions (11, 14). Moreover, vaccine effectiveness might also be considered by epidemiological parameters describing disease dynamics and a reduction in virus transmission (15, 16).

Literature on vaccination effectiveness in the field and the impact on production parameters is scarce (17). However, this information is needed for farmers to convert positive effects of vaccination into monetary values and to estimate financial benefits through economic assessments and gross margin analyses (18). Moreover, the benefit of a vaccine in relation to its cost and thus vaccine price and return on investment are determining factors that influence farmer's attitude and willingness to pay for a vaccine (19). When trying to estimate financial benefits on a national level, these are important aspects, which might affect vaccination coverage and the proportion of farmers adopting a new vaccine. With these preconditions, scenario modeling and stochastic simulations are common approaches used to account for missing information and uncertainty.

The aim of this study was to assess economic effects associated with vaccination against PRRS and to estimate financial benefits on animal, herd, and national level. To evaluate the impact of various vaccine characteristics, different levels of vaccination effectiveness, vaccine price, and vaccination coverage were modeled.

## Materials and Methods

### Model Description

The economic assessment of vaccination against PRRS is based on a stochastic model developed by Nathues et al. (3). The initial model estimated the financial burden of disease in endemically PRRSV-infected farms and served as baseline to model the economic efficiency of different control strategies against PRRS (6). To estimate the potential financial benefit of different vaccine characteristics and vaccination scenarios within this project, the initial model was adapted and expanded accordingly. The model was built in Excel (Microsoft Corporation, Redmont, Washington, USA) and the Add-on @Risk was used (Palisade Corporation, Newfield, New York, USA) to account for uncertainty and variability of parameters. The spreadsheet model consisted of several sub-models, which were linked with each other. Production parameters and epidemiological effects were identified and assessed in a literature review and implemented into the initial model. The production model simulated production dynamics within a timeframe of one year. The epidemiological flow model simulated disease impact according to defined disease status and incorporated these effects into the production model. In the intervention model, the cost and effectiveness associated with different vaccine characteristics and intervention strategies were defined and linked with the production and epidemiological model. The economic impact of disease and intervention was assessed by performing a gross margin analysis using production model outputs. The gross margin was defined as the total revenue minus variable cost. Variable cost consisted of replacement cost, market prices for sold or slaughtered animals, feeding cost, veterinary cost, cost for disposal of dead animals, variable energy cost, and miscellaneous cost. Full details of the baseline model, including prices of economic parameters, can be obtained from Nathues et al. (3) and the Supplementary Material. Stochastic simulations were run with 1,000 iterations per scenario. For the most part, stochastic model outputs were not normally distributed and estimates are, unless stated otherwise, reported as medians.

### Farm Description

To model economic effects at farm-level, a typical farrow-to-finish farm from a pig-dense region in Germany was chosen to serve as an example farm. As this farm type contains all three production stages (breeding, nursing, and fattening), it was possible to capture financial effects for each of the three production parts separately. Furthermore, this approach permits that the model could also be used for economic evaluations of farms that only have one or two of the production parts (i.e., breeding herd with the sale of weaners, breeding herd with the sale of nursery pigs, nursery only, fattening only). The example farm has 1,000 working sows, batch-wise farrowing every week, 3 weeks suckling period, an annual replacement rate of 35%, and finishers are sold at 120 kg live weight. A detailed description of farm characteristics is listed in Table 1. For the disease impact estimation, it was assumed that field virus was newly detected in a farm endemically infected with PRRSV. The farm previously did not vaccinate against PRRS and both reproduction and respiratory components of the disease were present in the herd. Consequently, it was expected that clinical effects would occur in all production parts along with reduced performance and production output in breeding, nursing, and fattening. Values of production parameters of a PRRS negative farm, a PRRSV-infected farm, and absolute disease effects are listed in Table 2. Negative farm data was obtained from industry reports (20, 21) whereas information on the magnitude of disease effects in endemically PRRSV-infected farms was assessed through an expert poll conducted within the study framework of the initial model (3). Out of various scenarios with different levels of disease severity described in the initial model, a moderate scenario with median values for clinical affectedness was selected to serve as basis for the conducted analyses.

**Table 1:** Farm characteristics of the example farrow-to-finish farm used for the economic model.

**Parameter**	**Value**
Number of working sows	1,000
Production rhythm (weeks)	3
Duration of suckling period (weeks)	3
Replacement rate (%)	35
Feed consumption during gestation (kg)	275
Feed consumption during lactation (kg)	200
Downtime between turns in nursery pens (days)	2
Downtime between turns in fattening pens (days)	5
Weight after nursery (kg)	30
Weight at slaughter (kg)	120

**Table 2:** Production parameters with assumed values of a PRRS negative farm and a PRRSV-infected farm with corresponding absolute disease effects.

**Parameter**	**Negative farm**	**Infected farm**	**Disease effect**
Return-to-oestrus rate	10.0%	13.5%	+3.5%
Abortion rate	2.0%	3.9%	+1.9%
Average piglets born alive per sow and litter	12.7	11.4	−1.3
Pre-weaning mortality	11.0%	13.5%	+2.5%
Weight at weaning	6.0 kg	5.5 kg	−0.5 kg
Days in nursery	45 days	50 days	+5 days
PRRS morbidity in weaners	–	20.0%	+20.0%
Mortality in weaners	3.0%	10.0%	+7.0%
Days in fattening	119	127 days	+8 days
PRRS morbidity in fatteners	–	20.0%	+20.0%
Mortality in fatteners	1.5%	3.0%	+1.5%

### Vaccination Strategies and Vaccine Characteristics

Two different vaccination strategies were considered in the model: (i) mass vaccination of sows only (MS) and (ii) mass vaccination of sows and vaccination of piglets (MSP). For MS, the vaccination protocol comprised a basic immunization of all sows and a booster vaccination 4 weeks later. After this, the entire sow herd is periodically vaccinated every 3 month. Incoming gilts are vaccinated twice during acclimatization. The MSP strategy is following the same protocol as the MS but with additional vaccination of piglets between the ages of 2–3 weeks.

The two main vaccine characteristics and associated economic effects examined in this study were vaccination effectiveness and vaccine price. For the economic modeling, the vaccination effectiveness was defined as the proportion by which disease effects in production parameters would be reduced after vaccination. Thus, related data on the improved of production output was required for implementation into the production model. The effectiveness of a vaccine when deployed in the field depends not only on the vaccine efficacy under ideal conditions but also on the characteristics of the population to whom it is administered and the comparison population (14). Since no evidence-based data was available, different levels of vaccination effectiveness were modeled: 50, 60, 70, 80, and 90%. In this context, an assumed vaccination effectiveness of 80% would mean the following: If the baseline abortion rate in a PRRS negative farm is 2% and in a PRRSV-infected farm 3.9%, the absolute disease effect is +1.9% (Table 1). Vaccination would reduce disease effects by 80% (−1.52%) and the abortion rate would persist at 2.38% after vaccination. To account for uncertainty and variation of vaccine price, several scenarios with different price levels were modeled: €0.75, 1.00, 1.25, and 1.50. The vaccine price was defined as the price per dose (including labor) for the single vaccination of one sow. For the MSP strategy, vaccination of a piglet would cost 80% of the price of sow vaccination.

### National-Level Analyses and Vaccination Coverage

The estimation of national-level benefits was based on outcomes from farm-level analyses. Demographic data on the pig population in Germany was obtained from the Federal Statistical Office (22). Overall, there are 27.2 million pigs, located on 23,800 farms. Of these, 8,400 farms hold breeding sows with totally 1.9 million sows. Herd-level prevalence show regional differences and is estimated to be between 50 and 100%, with high prevalence particularly in pig dense regions (23).

For the analyses, the breeding sow population was divided into two groups: previously vaccinated sows (preVS) and previously non-vaccinated sows (pnonVS). Previously vaccinated sows were defined as sows that were vaccinated against PRRS with a licensed and commercially available vaccine in the past. On the other hand, pnonVS were defined as sows that were not vaccinated against PRRS yet. The proportion of preVS is equivalent to the initial vaccination coverage whereas the proportion of pnonVS is equal to one minus the vaccination coverage. No empiric data on vaccination coverage was available and consequently different levels of preVS and pnonVS were modeled to account for uncertainty of this parameter. The proportion of sows that were vaccinated with a potential new vaccine (nVac) was independent from vaccination coverage and could be different in preVS and pnonVS. In preVS, it described the switch from a previously used licensed vaccine to nVac, whereas in pnonVS it described the switch from not vaccinating sows to newly vaccinating sows using nVac.

Different levels of vaccination effectiveness were modeled in the national-level analyses. For pnonVS, these comprised 50, 60, 70, 80, and 90%. For preVS, vaccination effectiveness of nVac was expressed as absolute change in effectiveness and five levels were considered (+5, +10, +15, +20, and +25%). For example, an effectiveness of +5% implied that disease effects were reduced by an additional 5%, compared to the previously used vaccine. To estimate financial benefits at national level, individual benefits per breeding sow were extrapolated. Animal-level benefits used as input values were obtained from farm-level analyses and were then multiplied by the corresponding number of affected animals. For national-level analyses, vaccine price was fixed at €1 for all scenarios.

## Results

**Figure 1:**
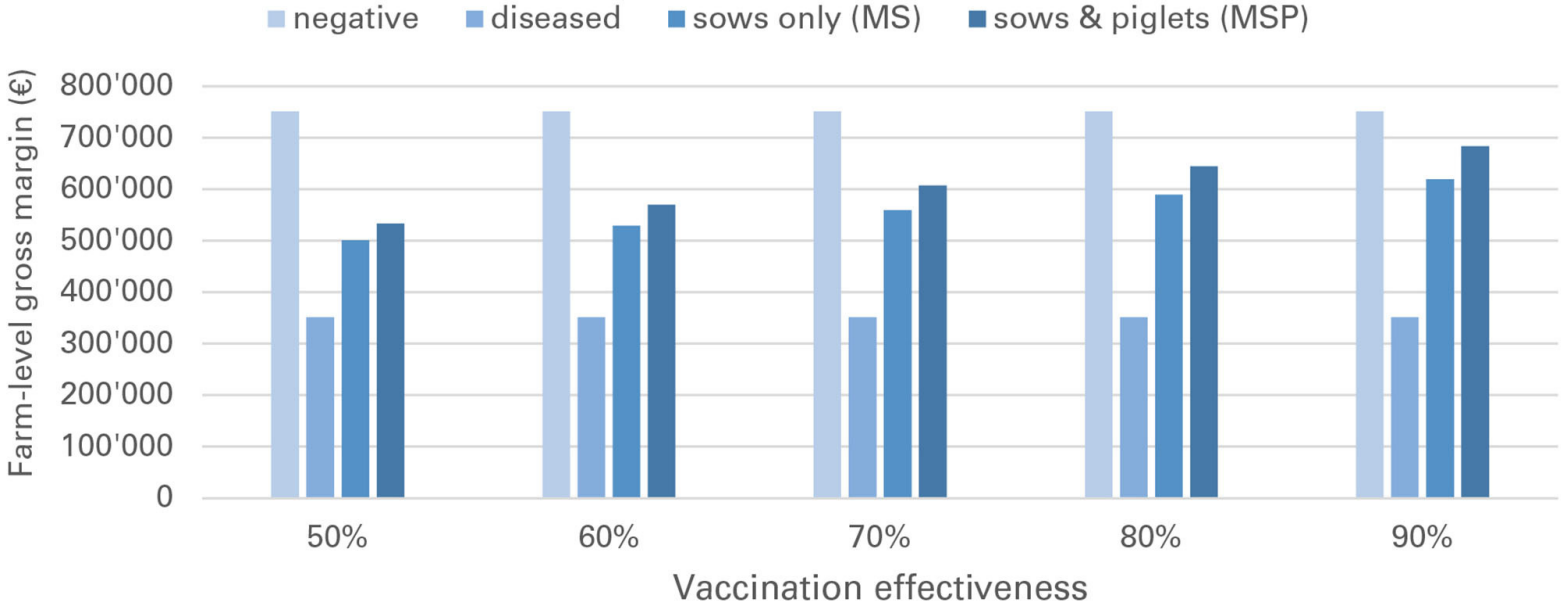
Annual gross margins for a PRRS negative farm, a PRRSV-infected farm without vaccination (“diseased”) and a PRRSV-infected farm with vaccination (MS and MSP strategy).

**Figure 2:**
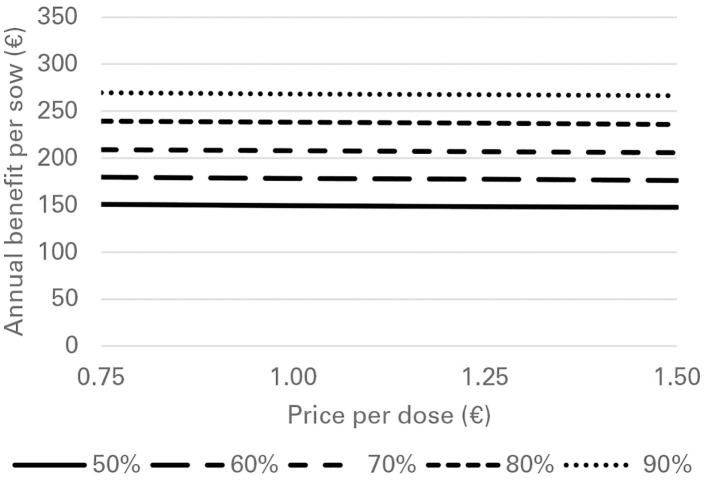
Annual benefit per working sow, depending on vaccination effectiveness, and vaccine price, when vaccinating sows only (MS strategy) against PRRS.

**Figure 3:**
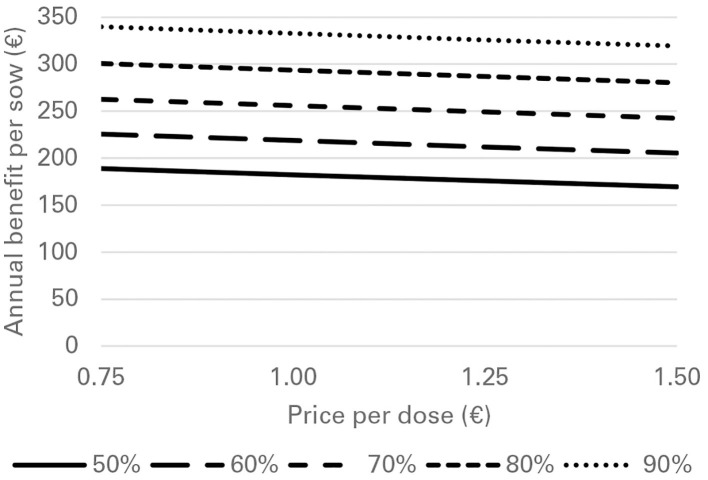
Annual benefit per working sow, depending on vaccination effectiveness, and vaccine price, when vaccinating sows and piglets (MSP strategy) against PRRS.

Financial losses due to PRRS, expressed as difference in gross margin between a PRRSV negative and a PRRSV-infected farrow-to-finish farm with disease effects according to Table 2 and 1,000 working sows were €400,018 per year (Figure 1). The median farm-level financial benefit attributable to vaccination with nVac ranged from €147,525 to 269,759 when sows only are vaccinated (Table 3) and from €169,563 to 339,643 in the MSP strategy, when piglets are also vaccinated (Table 4). Consequently, annual animal-level benefits per working sow attributable to vaccination were estimated to be between €148 and 270 for MS (Figure 2) and between €170 and 340 for MSP (Figure 3). These two figures give an overview of the associations between the modeled vaccine characteristics and financial benefits per working sow. The magnitude of financial benefits generated was more sensitive to a change in vaccination effectiveness than the variation of vaccine price. Particularly in the MS strategy, vaccine price had only minor effects on the profitability, whereas effects were more pronounced when piglets are vaccinated as well. For all modeled scenarios, the MSP strategy was more beneficial than MS when identical vaccine characteristics were assumed.

**Table 3:** Differences in annual gross margin (in €) between a PRRSV-infected farm without intervention and a PRRS-infected farm with mass vaccination of sows (MS).

**Vaccine price**	**Vaccination effectiveness**				
	**50%**	**60%**	**70%**	**80%**	**90%**
€0.75	150′727	179′865	209′149	239′455	269′759
€1.00	149′581	178′387	208′127	238′336	268′272
€1.25	148′313	177′482	207′020	237′136	267′480
€1.50	147′525	176′312	205′959	235′820	266′435

**Table 4:** Differences in annual gross margin (in €) between a PRRSV-infected farm without intervention and a PRRSV-infected farm with mass vaccination of sows and piglets (MSP).

**Vaccine price**	**Vaccination effectiveness**				
	**50%**	**60%**	**70%**	**80%**	**90%**
€0.75	188′938	225′481	262′646	300′798	339′643
€1.00	182′137	218′858	255′985	293′787	332′766
€1.25	175′883	211′992	249′089	287′053	325′839
€1.50	169′563	205′473	242′660	280′265	319′222

Financial benefits at national level were generated by adopting nVac to both preVS and pnonVS. Outputs were dependent on the variation of vaccination effectiveness, vaccination coverage, and the proportion of sows newly vaccinated with nVac.

**Figure 4:**
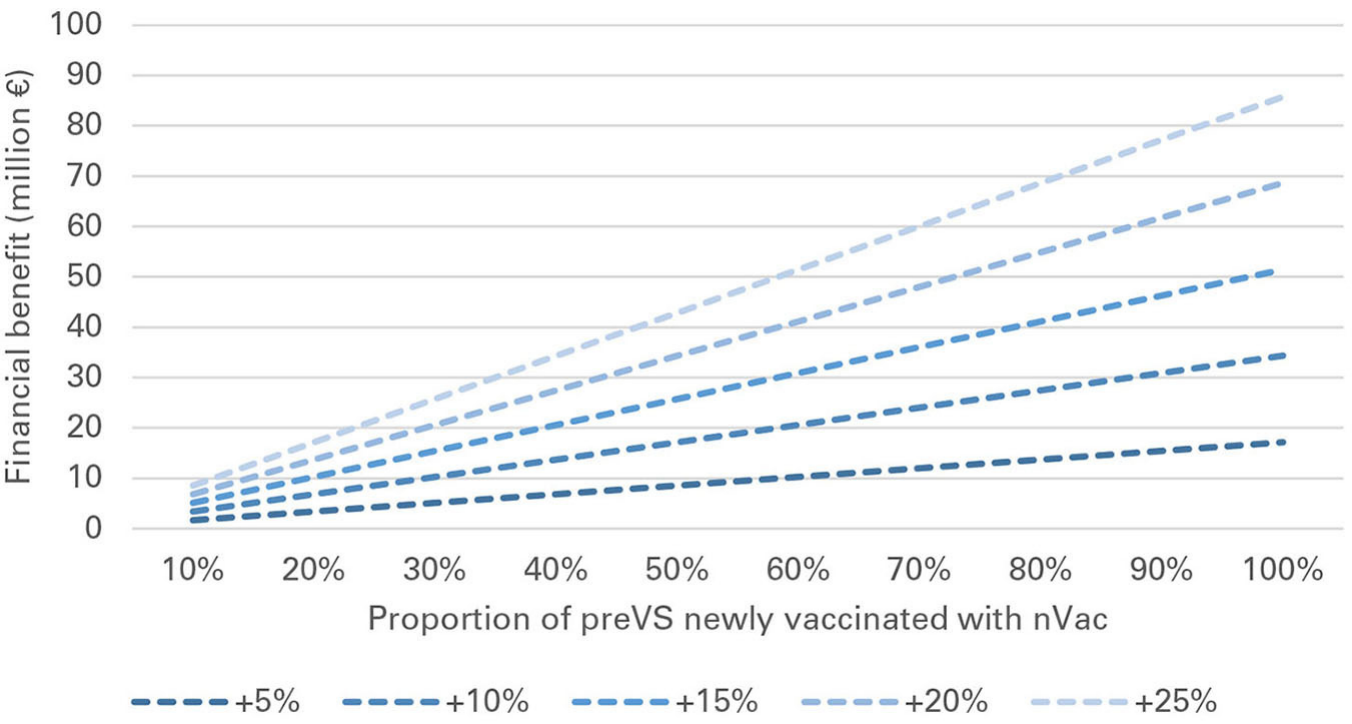
Financial benefit associated with vaccination of previously vaccinated sows (preVS), when vaccination coverage is 60%, and vaccination effectiveness of nVac ranges from +5 to +25%.

In preVS, for each sow newly vaccinated with nVac, a median benefit of €3 was gained for every +1% of increased effectiveness compared with the effectiveness of the previously used vaccine. National-level financial benefits ranged from €2.3 to 45.7 million for the listed scenarios and a fixed vaccination effectiveness of +10% (Table 5). As an example, if 30% of sows were newly vaccinated with nVac and vaccination coverage was 60%, the annual financial benefit was estimated to be €10.3 million. If vaccination coverage was fixed at 60% and vaccination effectiveness varied from +5 to +25%, the financial benefit ranged from €1.7 to 85.8 million (Table 6). The proportion of sows newly vaccinated with nVac had a substantial effect on the magnitude of financial benefit, especially when effectiveness increased (Figure 4).

**Table 5:** Financial benefits (in €) associated with vaccination of previously vaccinated sows (preVS), when vaccination effectiveness of a potential new vaccine (nVac) is +10% compared to the previously used alternative vaccine.

**Proportion of nVac in preVS**	**Vaccination coverage**				
	**40%**	**50%**	**60%**	**70%**	**80%**
10%	2′287′440	2′859′300	3′431′160	4′003′020	4′574′880
20%	4′574′880	5′718′600	6′862′320	8′006′040	9′149′760
30%	6′862′320	8′577′900	10′293′480	12′009′060	13′724′640
40%	9′149′760	11′437′200	13′724′640	16′012′080	18′299′520
50%	11′437′200	14′296′500	17′155′800	20′015′100	22′874′400
60%	13′724′640	17′155′800	20′586′960	24′018′120	27′449′280
70%	16′012′080	20′015′100	24′018′120	28′021′140	32′024′160
80%	18′299′520	22′874′400	27′449′280	32′024′160	36′599′040
90%	20′586′960	25′733′700	30′880′440	36′027′180	41′173′920
100%	22′874′400	28′593′000	34′311′600	40′030′200	45′748′800

*Estimates depend on vaccination coverage and the proportion of preVS that are newly vaccinated with nVac*.

**Table 6:** Financial benefits (in €) due to vaccination of previously vaccinated sows (preVS) with nVac instead of a previously used licensed vaccine (vaccination coverage fixed at 60%).

**Proportion of nVac in preVS**	**Vaccination effectiveness**				
	**+5%**	**+10%**	**+15%**	**+20%**	**+25%**
10%	1′715′580	3′431′160	5′146′740	6′862′320	8′577′900
20%	3′431′160	6′862′320	10′293′480	13′724′640	17′155′800
30%	5′146′740	10′293′480	15′440′220	20′586′960	25′733′700
40%	6′862′320	13′724′640	20′586′960	27′449′280	34′311′600
50%	8′577′900	17′155′800	25′733′700	34′311′600	42′889′500
60%	10′293′480	20′586′960	30′880′440	41′173′920	51′467′400
70%	12′009′060	24′018′120	36′027′180	48′036′240	60′045′300
80%	13′724′640	27′449′280	41′173′920	54′898′560	68′623′200
90%	15′440′220	30′880′440	46′320′660	61′760′880	77′201′100
100%	17′155′800	34′311′600	51′467′400	68′623′200	85′779′000

**Figure 5:**
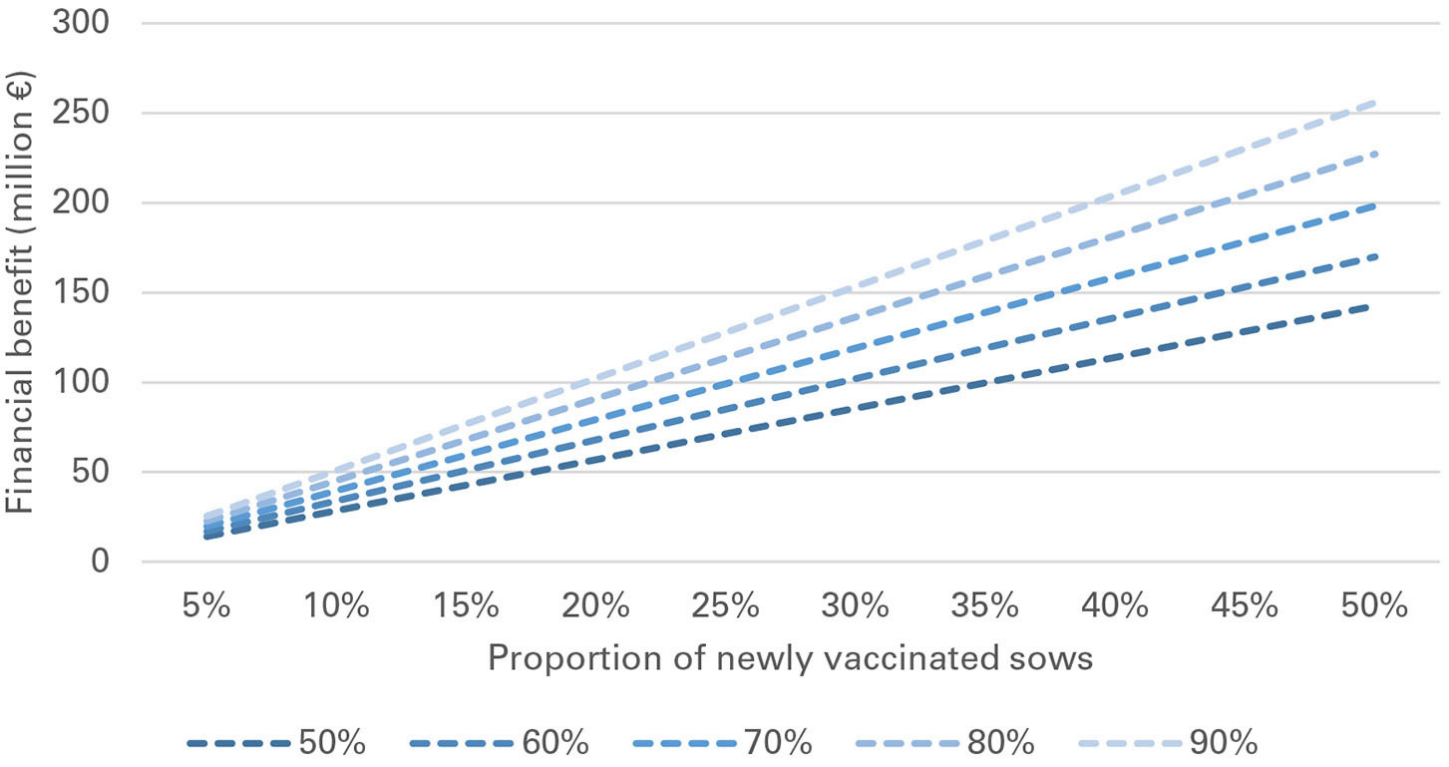
Financial benefit associated with vaccination of previously non-vaccinated sows (pnonVS) for different levels of vaccination effectiveness and proportions of newly vaccinated sows.

In pnonVS, financial benefits per sow were substantially higher than in preVS. Median benefits per sow ranged from €150 to 268, depending on vaccination effectiveness and at a fixed vaccine price of €1. Already a small number of pnonVS newly vaccinated with nVac generated significant benefits at national level. If vaccination effectiveness varied between 50 and 90% and between 1 and 10% of sows were vaccinated with nVac, financial benefits ranged from €2.9 to 51.1 million (Table 7). If initial vaccination coverage was 50% and all of the pnonVS would newly be vaccinated with nVac, financial benefits would range from €143 to 256 million, depending on vaccination effectiveness (Figure 5).

**Table 7:** Financial benefits (in €) associated with vaccination of previously non-vaccinated sows (pnonVS) for different levels of vaccination effectiveness and proportion of newly vaccinated sows.

**Proportion of sows vaccinated with nVac**	**Vaccination effectiveness**				
	**50%**	**60%**	**70%**	**80%**	**90%**
1%	2′851′317	3′400′409	3′967′309	4′543′155	5′113′805
2%	5′702′634	6′800′818	7′934′618	9′086′310	10′227′609
3%	8′553′951	10′201′228	11′901′928	13′629′465	15′341′414
4%	11′405′267	13′601′637	15′869′237	18′172′620	20′455′219
5%	14′256′584	17′002′046	19′836′546	22′715′776	25′569′023
6%	17′107′901	20′402′455	23′803′855	27′258′931	30′682′828
7%	19′959′218	23′802′864	27′771′165	31′802′086	35′796′633
8%	22′810′535	27′203′273	31′738′474	36′345′241	40′910′437
9%	25′661′852	30′603′683	35′705′783	40′888′396	46′024′242
10%	28′513′168	34′004′092	39′673′092	45′431′551	51′138′047

## Discussion

Results from this study demonstrate that the introduction of nVac is associated with substantial financial benefits at animal, farm, and national level.

When interpreting farm-level outcomes it has to be considered that the estimated benefits are reported as differences in gross margin between a PRRSV-infected herd without previous intervention and a PRRSV-infected herd with intervention, in the form of vaccination. Furthermore, it was assumed that all production parts were affected by PRRS, which was associated with substantially reduced production performance and farm output and thus farm revenue. Therefore, it has to be taken into account that in practice it is likely that a farm showing such magnitudes of disease effects would already have implemented some sort of disease control, possibly in the form of vaccination with an already licensed and commercially available vaccine. This would imply that financial benefits of using nVac would have to be obtained from comparing farms vaccinating with nVac and farms vaccinating with an “old” vaccine. Consequently, reported estimates represent the maximum possible benefits based on the situation when no previous disease control was established. Financial benefits in farms that switch from an alternative vaccine to nVac are less pronounced, as demonstrated in the national-level models.

Estimates from this study are based on an example farm where both reproductive and respiratory traits were affected by PRRS. The reproduction parameters “abortion rate” and “number of piglets born alive” had a significant impact on gross margin. When an increased number of piglets are born due to improved reproductive performance after vaccination, more pigs go through the different production stages and eventually more fatteners are sold for slaughter. This had a direct impact on the farm output and consequently led to higher farm revenue. A main effect of the respiratory component of the disease is the reduced performance of growing pigs (24, 25). This effect is also reflected in the finding that the MSP strategy is more profitable than the MS. The additional cost for the piglet vaccination was compensated and surpassed by the improved production performance in the nursery and fattening stage.

The sensitivity analysis showed that the gross margin and associated economic profitability were more sensitive to vaccination effectiveness than to vaccine price. However, the range of vaccine prices included in the scenarios was limited. A potential novel vaccine stands in competition with other PRRS vaccines (7) and therefore its price would be influenced by the price of the already commercially available vaccines. Furthermore, it is likely that vaccination effectiveness and vaccine price are correlated. If vaccination effectiveness is high, farmers might be willing to pay more and when farmers can chose between different products, they would only purchase vaccines that are more expensive if vaccination effectiveness is increased accordingly (26). Overall, a “vaccine cost-benefit ratio,” expressed as vaccine price compared to its vaccination effectiveness, could serve as key criteria in farmer's decision-making process. This implies that even if the vaccination effectiveness of nVac would be lower than the effectiveness of alternative vaccines, farmers might still switch to nVac under certain circumstances. Consequently, setting a market price for nVac without knowing its effectiveness in the field would be associated with a high level of uncertainty and thus different scenarios were modeled. In addition, market prices of vaccines do not only depend on its effectiveness alone, but do include many other criteria and are defined in a multi-step procedure (27) which implies that vaccine prices used in this model do not permit conclusions about production cost or margins of the vaccine manufacturer.

The results indicate that benefits generated in pnonVS are significantly higher than in preVS. This is because infected pnonVS showed substantially reduced production performance prior to vaccination whereas disease effects in preVS were already diminished due to vaccination with an alternative vaccine. However, the contribution to the overall financial benefit might still be larger from preVS than from pnonVS. It is likely that in practice, the proportion of sows newly vaccinated with nVac might be substantially higher in preVS than in pnonVS. It might be more challenging to convince farmers to start vaccinating their sows against PRRS (and chose nVac) than convincing farmers that already have been vaccinating their sows to switch to nVac. These farmers are already familiar with PRRS vaccination, associated cost, labor, and equipment and thus might be more likely to start using a new vaccine (28). A further point for discussion is the impact of nVac on the market equilibrium, associated with reduced disease prevalence (29). If a large proportion of farmers were vaccinating their sows, production performance would increase, more pigs would be marketed and eventually supply increases. This would lead to a shift of the supply curve and a change of market equilibrium associated with lower prices for farmers (30). However, market impacts were not considered in this study.

In the context of scenario analysis, reported financial benefits for different scenarios modeled in this study do not consider the plausibility of the corresponding scenario. The likelihood of a certain scenario has to be further investigated and supported with empiric data as no information on current vaccination coverage or public data on market shares of existing PRRS vaccines is available. Moreover, without yet knowing vaccination effectiveness and vaccine price, it is difficult to estimate to what extent nVac would be adopted in the field. The proportion of farmers that would switch from an alternative vaccine to nVac, as well as the proportion of farmers who would newly start vaccinating against PRRS, significantly depend on these key vaccine characteristics. Therefore, potential socio-economic benefits largely depend on farmers' attitude toward PRRS vaccination and their decisions with respect to switching to a new vaccine (31). The behavior of a few single farmers might have a significant impact at national level, depending on their farm size. In Germany, farm sizes are rather heterogeneous (22). There are many small farms and only few large pig farms. When looking at breeding farms, 24% of them have <50 sows but account for only 2% of the population. On the other hand, only 8% of farms have more than 500 sows but make up for 42% of all breeding sows in Germany. This implies that if a few owners of large farms decide to switch to nVac, a considerable number of sows are affected. However, the decision to switch to nVac might not only be a farmers' own choice. The decision-making process might also be influenced and controlled by a number of external factors (28).

## Conclusions

The analysis performed showed that while vaccination against PRRS was beneficial for all modeled scenarios, vaccinating both sows and piglets proved to be more profitable than vaccinating sows alone. The magnitude of benefits derived from vaccination was more sensitive to changes in vaccine effectiveness than to vaccine price changes. Actual economic benefits of nVac largely depend on the extent to which it would be adopted in the field and consequently also on farmer's attitude toward a new vaccine and willingness to pay.

## Data Availability Statement

The original contributions presented in the study are included in the article/supplementary material, further inquiries can be directed to the corresponding author/s.

## Author Contributions

HN, JR, BT, and GS-R contributed to the conception and design of the study. BT developed the model and run the analyses. BT and HN evaluated results and discussed their implications. BT wrote a first draft of the manuscript. All authors contributed to the manuscript revision, read, and approved the submitted version.

## Conflict of Interest

The authors declare that the research was conducted in the absence of any commercial or financial relationships that could be construed as a potential conflict of interest.

## References

[1] Nieuwenhuis N Duinhof TF Van Nes A. Papers: economic analysis of outbreaks of porcine reproductive and respiratory syndrome virus in nine sow herds. Vet Rec. (2012) 170:225. 10.1136/vr.100101 22238201

[2] Holtkamp DJ Kliebenstein JB Neumann EJ Zimmerman JJ Rotto HF Yoder TK. Assessment of the economic impact of porcine reproductive and respiratory syndrome virus on United States pork producers. J Swine Heal Prod. (2013) 21:72–84. 10.31274/ans_air-180814-2816121604

[3] Nathues H Alarcon P Rushton J Jolie R Fiebig K Jimenez M. Cost of porcine reproductive and respiratory syndrome virus at individual farm level - an economic disease model. Prev Vet Med. (2017) 142:16–29. 10.1016/j.prevetmed.2017.04.006 28606362

[4] Neumann EJ Kliebenstein JB Johnson CD Mabry JW Bush EJ Seitzinger AH. Assessment of the economic impact of porcine reproductive and respiratory syndrome on swine production in the United States. J Am Vet Med Assoc. (2005) 227:385–92. 10.2460/javma.2005.227.385 16121604

[5] Lunney JK Benfield DA Rowland RRR Porcine reproductive and respiratory syndrome virus: an update on an emerging and re-emerging viral disease of swine. Virus Res. (2010) 154:1–6. 10.1016/j.virusres.2010.10.009 20951175 PMC7172856

[6] Nathues H Alarcon P Rushton J Jolie R Fiebig K Jimenez M. Modelling the economic efficiency of using different strategies to control porcine reproductive & respiratory syndrome at herd level. Prev Vet Med. (2018) 152:89–102. 10.1016/j.prevetmed.2018.02.005 29559110

[7] Nan Y Wu C Gu G Sun W Zhang YJ Zhou EM. Improved vaccine against PRRSV: current progress and future perspective. Front Microbiol. (2017) 8:1635. 10.3389/fmicb.2017.01635 28894443 PMC5581347

[8] Bernelin-Cottet C Urien C Fretaud M Langevin C Trus I Jouneau L A DNA prime immuno-potentiates a modified live vaccine against the porcine reproductive and respiratory syndrome virus but does not improve heterologous protection. Viruses. (2019) 11:576 10.3390/v11060576 PMC6631340 31242645

[9] Bernelin-Cottet C Urien C Stubsrud E Jakob V Bouguyon E Bordet E. A DNA-modified live vaccine prime-boost strategy broadens the T-cell response and enhances the antibody response against the porcine reproductive and respiratory syndrome virus. Viruses. (2019) 11:551. 10.3390/v11060551 31207934 PMC6630347

[10] Kimman TG Cornelissen LA Moormann RJ Rebel JMJ Stockhofe-Zurwieden N. Challenges for porcine reproductive and respiratory syndrome virus (PRRSV) vaccinology. Vaccine. (2009) 27:3704–18. 10.1016/j.vaccine.2009.04.022 19464553

[11] Canelli E Catella A Borghetti P Ferrari L Ogno G De Angelis E. Efficacy of a modified-live virus vaccine in pigs experimentally infected with a highly pathogenic porcine reproductive and respiratory syndrome virus type 1 (HP-PRRSV-1). Vet Microbiol. (2018) 226:89–96. 10.1016/j.vetmic.2018.10.001 30389048

[12] Renukaradhya GJ Meng XJ Calvert JG Roof M Lager KM. Inactivated and subunit vaccines against porcine reproductive and respiratory syndrome: current status and future direction. Vaccine. (2015) 33:3065–72. 10.1016/j.vaccine.2015.04.102 25980425

[13] Bonckaert C van der Meulen K Rodríguez-Ballarà I Pedrazuela Sanz R Martinez MF Nauwynck HJ. Modified-live PRRSV subtype 1 vaccine UNISTRAIN® PRRS provides a partial clinical and virological protection upon challenge with East European subtype 3 PRRSV strain Lena. Porc Heal Manag. (2016) 2:12. 10.1186/s40813-016-0029-y 28405438 PMC5382438

[14] Comstock GW. Evaluating vaccination effectiveness and vaccine efficacy by means of case-control studies. Epidemiol Rev. (1994) 16:77–89. 10.1093/oxfordjournals.epirev.a036147 7925730

[15] Bitsouni V Lycett S Opriessnig T Doeschl-Wilson A. Predicting vaccine effectiveness in livestock populations: a theoretical framework applied to PRRS virus infections in pigs. PLoS ONE. (2019) 14:e0220738. 10.1371/journal.pone.0220738 31469850 PMC6716781

[16] Chase-Topping M Xie J Pooley C Trus I Bonckaert C Rediger K. New insights about vaccine effectiveness: Impact of attenuated PRRS-strain vaccination on heterologous strain transmission. Vaccine. (2020) 38:3050–61. 10.1016/j.vaccine.2020.02.015 32122719

[17] Moura CAA Johnson C Baker SR Holtkamp DJ Wang C Linhares DCL. Assessment of immediate production impact following attenuated PRRS type 2 virus vaccination in swine breeding herds. Porc Heal Manag. (2019) 5:13. 10.1186/s40813-019-0120-2 31183160 PMC6542134

[18] Rushton J Thornton PK Otte MJ. Methods of economic impact assessment. Rev Sci Tech. (1999) 18:315–42. 10.20506/rst.18.2.1172 10472671

[19] Bennett R Balcombe K Farmers' willingness to pay for a tuberculosis cattle vaccine. J Agric Econ. (2012) 63:408–24. 10.1111/j.1477-9552.2011.00330.x

[20] Anonymous Schweineproduktion 2011 in Deutschland. 20th ed Bonn: Zentralverband der Deutschen Schweineproduktion (ZDS) (2012).

[21] Anonymous Interpig - 2013 Pig Cost of Production in Selected Countries. AHDB. (2014). Available online at: https://pork.ahdb.org.uk/media/2371/2013_pig_cost_of_production_in_selected_countries.pdf

[22] Destatis Viehbestand und Tierische Erzeugung 2017 - Fachserie 3, Reihe 4. Statistisches Bundesamt. (2018). Available online at: https://www.destatis.de/DE/Publikationen/Thematisch/LandForstwirtschaft/ViehbestandTierischeErzeugung/ViehbestandtierischeErzeugung.html

[23] Ritzmann M Stadler J Adam L Möglichkeiten und Grenzen der PRRS-Eliminierung. In: Rackwitz R Pees M Aschenbach JR Gäbel G, editors. LBH: 8. Leipziger Tierärztekongress - Tagungsband 3. University of Leipzig, Leipzig. Berlin: Lehmanns Media GmbH (2016). p. 206–7.

[24] Helm ET Curry SM De Mille CM Schweer WP Burrough ER Zuber EA. Impact of porcine reproductive and respiratory syndrome virus on muscle metabolism of growing pigs. J Anim Sci. (2019) 97:3213–27. 10.1093/jas/skz168 31212312 PMC6667233

[25] Schweer WP Schwartz K Burrough ER Yoon KJ Sparks JC Gabler NK The effect of porcine reproductive and respiratory syndrome virus and porcine epidemic diarrhea virus challenge on growing pigs I: Growth performance and digestibility1. J Anim Sci. (2016) 94:514–22. 10.2527/jas.2015-9834 27065121 PMC7199662

[26] Wane A Dione M Wieland B Rich KM Yena AS Fall A. Willingness to vaccinate (WTV) and willingness to pay (WTP) for vaccination against peste des petits ruminants (PPR) in Mali. Front Vet Sci. (2020) 6:488. 10.3389/fvets.2019.00488 32010711 PMC6974520

[27] Lee BY McGlone SM Pricing of new vaccines. Hum Vaccin. (2010) 6:619–26. 10.4161/hv.6.8.11563 20861678 PMC3056061

[28] Burchett HED Mounier-Jack S Griffiths UK Mills AJ. National decision-making on adopting new vaccines: a systematic review. Health Policy Plan. (2012) 27(Suppl 2):ii62–76. 10.1093/heapol/czr049 21733989

[29] Johnson KK Pendell DL. Market impacts of reducing the prevalence of bovine respiratory disease in United States beef cattle feedlots. Front Vet Sci. (2017) 4:189. 10.3389/fvets.2017.00189 29170739 PMC5684707

[30] Feng S Patton M Davis J. Market impact of foot-and-mouth disease control strategies: a UK case study. Front Vet Sci. (2017) 4:129. 10.3389/fvets.2017.00129 28920059 PMC5585142

[31] Barratt AS Rich KM Eze JI Porphyre T Gunn GJ Stott AW. Framework for estimating indirect costs in animal health using time series analysis. Front Vet Sci. (2019) 6:190. 10.3389/fvets.2019.00190 31275949 PMC6592220

