# Supplementary material for: Modeling Economic Effects of Vaccination Against Porcine Reproductive and Respiratory Syndrome: Impact of Vaccination Effectiveness, Vaccine Price, and Vaccination Coverage
Source: Front Vet Sci. 2020 Aug 11;7:500. doi: 10.3389/fvets.2020.00500 (PMC7431490; doi:10.3389/fvets.2020.00500)
Supplement: Supplementary file 1 [file Table_1.pdf]

Table S1. Economic parameters of a typical farrow-to-finish herd with 1,000 sows in Northern Germany used for the production model.

Parameter	Value (€)	Reference/Calculation
**Pig prices**		
Price per sow slaughtered	220	(1)
Price of a replacement gilt	350	(2)
Price per kg live weight of a piglet sold at weaning	3.7	(1)
Price per kg live weight of a weaner sold	1.8	(1)
Price per kg live weight of a fattener sold	1.2	(1)
**Feed and water prices**		
Feed price/tonne (gestation)	230	(2,3)
Feed price/tonne (lactation)	280	(2,3)
Feed price/tonne (nursery)	320	(2,3)
Feed price/tonne (fatteners)	280	(3,4)
Water cost per litre	0.005	(5)
Veterinary costs		
Total veterinary cost per sow per year (incl. costs for piglets)	80	(1)
Total veterinary cost per weaner produced	2	(6)
Total veterinary cost per fattening pig	1.3	(4)
**Dead pig disposal prices**		
Disposal cost per kg animal disposed	0.15	(7)
**Insemination prices**		
Price per semen dose	3	(8)
**Transport prices**		
Transport costs for slaughter sows per sow	5	(2)
Transport cost per kg live weight pig	0.02	(2)
**Energy prices (incl. water except drinking water for animals)**		
Energy cost per sow and year	75	(2)
Energy cost per weaner produced	0.75	(6)
Energy cost per fattener produced	2.5	(4)
**Fixed cost prices**		
Labour cost breeding per year	200,000	(2)
Labour cost nursery per year	25,000	(6)
Labour cost fattening per year	120,000	(4)
Building cost breeding per year	200,000	(2)
Building cost nursery per year	60,000	(6)
Building cost fattening per year	100,000	(4)
Equipment cost breeding per year	5,000	(2)
Equipment cost nursery per year	25,000	(6)
Equipment cost fattening per year	50,000	(4)
Inspection, levy and insurance cost breeding per year	12,000	(2)
Inspection, levy and insurance cost nursery per year	12,000	(6)
Inspection, levy and insurance cost fattening per year	25,000	(4)
Any other fixed cost breeding per year	30,000	(2)
Any other fixed cost nursery per year	20,000	(2)
Any other fixed cost fattening per year	30,000	(4)

References:

1. Anonymous. Schlachtschweinepreis. *Erzeugergemeinschaft für Qual Hümmling eG* (2015) Available at: http://www.ezg-huemmling.de/marktberichte/schlachtschweine/schlachtschweinepreis.html [Accessed June 24, 2015]

2. Anonymous. Deckungsbeiträge und Kalkulationsdaten - Ferkelerzeugung. *LfL* (2015) Available at: https://www.stmelf.bayern.de/idb/ferkelerzeugungkonv.html [Accessed June 22, 2015]

3. Anonymous. Interpig - 2013 Pig Cost of Production in Selected Countries. *AHDB* (2014) Available at: https://pork.ahdb.org.uk/media/2371/2013_pig_cost_of_production_in_selected_countries.pdf

4. Anonymous. Deckungsbeiträge und Kalkulationsdaten – Schweinemast. *LfL* (2015) Available at: https://www.stmelf.bayern.de/idb/schweinemastkonv.html [Accessed June 23, 2015]

5. Anonymous. 1000 Liter Trinkwasserkosteten 2013 im Durchschnitt 1,69 Euro. *Pressemitteilungen* (2014) Available at: https://www.destatis.de/DE/PresseService/Presse/Pressemitteilungen/2014/03/PD14 110 322.html [Accessed February 28, 2015]

6. Gross D. Kosten und Rentabilität der Ferkelaufzucht. *DLR Westerwald-Osteifel* (2009) Available at: http://www.dlr-westerwald-osteifel.rlp.de/Internet/global/themen.nsf/63e1cb721c81af07c125706e004a2bad/8fffe42d64498e63c1256fe30033a25f?OpenDocument

7. Anonymous. Die Kosten der Tierkörperbeseitigung. *Hessische Tierseuchenkasse* (2015) Available at: http://www.hessischetierseuchenkasse.de/04_03_tierkoerperbeseitigung.html [Accessed February 25, 2015]

8. Anonymous. Preisliste Besamungen. *Schweinebesamungsstation Weser-Ems e.V.* (2015) Available at: http://www.schweinebesamung.de/preise.php [Accessed March 6, 2015]
